# Development of an electrooculogram-based human-computer interface using involuntary eye movement by spatially rotating sound for communication of locked-in patients

**DOI:** 10.1038/s41598-018-27865-5

**Published:** 2018-06-22

**Authors:** Do Yeon Kim, Chang-Hee Han, Chang-Hwan Im

**Affiliations:** Department of Biomedical Engineering, Hanyang University, Seoul, 04763 Republic of Korea

## Abstract

Individuals who have lost normal pathways for communication need augmentative and alternative communication (AAC) devices. In this study, we propose a new electrooculogram (EOG)-based human-computer interface (HCI) paradigm for AAC that does not require a user’s voluntary eye movement for binary yes/no communication by patients in locked-in state (LIS). The proposed HCI uses a horizontal EOG elicited by involuntary auditory oculogyric reflex, in response to a rotating sound source. In the proposed HCI paradigm, a user was asked to selectively attend to one of two sound sources rotating in directions opposite to each other, based on the user’s intention. The user’s intentions could then be recognised by quantifying EOGs. To validate its performance, a series of experiments was conducted with ten healthy subjects, and two patients with amyotrophic lateral sclerosis (ALS). The online experimental results exhibited high-classification accuracies of 94% in both healthy subjects and ALS patients in cases where decisions were made every six seconds. The ALS patients also participated in a practical yes/no communication experiment with 26 or 30 questions with known answers. The accuracy of the experiments with questionnaires was 94%, demonstrating that our paradigm could constitute an auxiliary AAC system for some LIS patients.

## Introduction

There are many individuals who lose normal pathways for communication. Patients with amyotrophic lateral sclerosis (ALS) are representative examples of the so-called ‘locked-in state (LIS)’ patients. ALS is a neurodegenerative disease that is characterised by progressive degeneration that causes death of motor neurons in brain and spinal cord. As the disease progresses, the brain gradually loses its ability to initiate action potentials to control voluntary body movements. Since the disease affects voluntary muscles, communication skills rapidly deteriorate. Therefore, individuals with ALS may need specific forms of assistive devices that can restore their speech intelligibility^1,2^.

Numerous studies have presented approaches to assist communication for patients with ALS. Augmentative and alternative communication (AAC) may be the most effective and appropriate tool that offers replacement of verbal and gestural communication in these cases. AAC includes a wide range of communication methods, ranging from low-technology methods, such as gestures, eye blink scanning, and communication boards, to high-technology methods, including advanced electronic devices for speech, typing, and web browsing^3–5^. Over recent years, performance and applicability of AAC technologies have been improved markedly. Accordingly, numerous number of ALS patients use AAC until the very late stages of the disease^4^.

Commonly used AAC systems, specifically for people with ALS, can be divided into two categories: 1) bio-signal-independent systems, and 2) bio-signal-dependent systems. A camera-based eye-tracking device is one of the most promising bio-signal-independent systems. It seamlessly tracks eye gaze movement as a user observes a visual screen, and allows computer access, or environmental control, with relatively high-classification accuracies^4,6,7^. However, there are still a few issues that need to be resolved, such as its high cost for daily use and the camera calibration requirements before each use^6,8,9^.

Some human-computer interfaces (HCIs) use a variety of bio-signals generated from our body to control external devices, or to translate an individual’s intention^10,11^. Physically impaired people can gain enormous benefits from such assistive devices, which enable them to maintain their communication function. In particular, a brain-computer interface (BCI) is a form of HCI that uses brain signals for communication and control, and has been actively studied in people with late-stage ALS. P300 event-related potentials (ERP)^12–15^, and steady-state visual evoked potentials (SSVEP)^12,16,17^ are two of the most extensively studied electroencephalography (EEG) signals for non-muscular communications in patients with ALS^12,18,19^. Both BCI paradigms use visual stimuli to elicit specific brain electrical responses. These can be potentially applied to a number of late-stage ALS patients since oculomotor function is generally the only remaining motor-related function to most late-stage ALS patients.

Despite the recent advancements in EEG-based BCI systems that use brain responses to visual stimuli, these systems still face some difficulties in being used into the majority of clinical or practical applications^20–23^. One of the critical limitations of traditional, visual-based BCI paradigms, is the basic assumption that the BCI users have an ability to voluntarily control their eye movements, and fix their eye gaze at a specific target location for a few seconds^24^. If BCI users do not meet these fundamental requirements, such as the case of patients with impaired eye movements, the visual-based BCI systems may not work effectively^21,25^. For instance, Averbuch-Heller *et al*. (1998) reported a case study of an ALS patient with slow vertical eye movements. The patient was able to move his eyes horizontally, but had difficulty in opening the eyelids and moving his eyes in the upward direction^26^. Another case study was that of a female ALS patient who had a sutured left eye to avoid double vision and was only able to move her eye vertically, who reported that the performance of a visual-based BCI system was not as high as those of other BCI systems, based on auditory and tactile stimuli^27^. These examples suggest that a variety of AAC systems still need to be developed to provide a specific ALS patient with the most appropriate communication options.

Even though electrooculogram (EOG)-based AAC devices^28,29^ have not been extensively developed, in contrast to other modalities, they may provide possible solutions for the current limitations of visual-based BCI systems. EOG signals yield high signal-to-noise ratio (SNR) values^30^, relatively simple waveforms, and a linear relationship with eye movements^31^. In comparison with EEG, EOG can be acquired using a fewer number of electrodes. Unlike camera-based eye tracking devices, the EOG-based AAC devices do not need additional systems (e.g. infrared cameras) except for a signal amplifier^32^. Despite these advantages of EOG-based AAC systems, however, the current EOG-based systems require the user’s voluntary movement of eyeballs. It seems obvious that patients with oculomotor impairment would require more efforts to voluntarily control eyeballs compared to performing simple mental tasks, e.g. directing concentration towards a target sound source.

**Figure 1:**
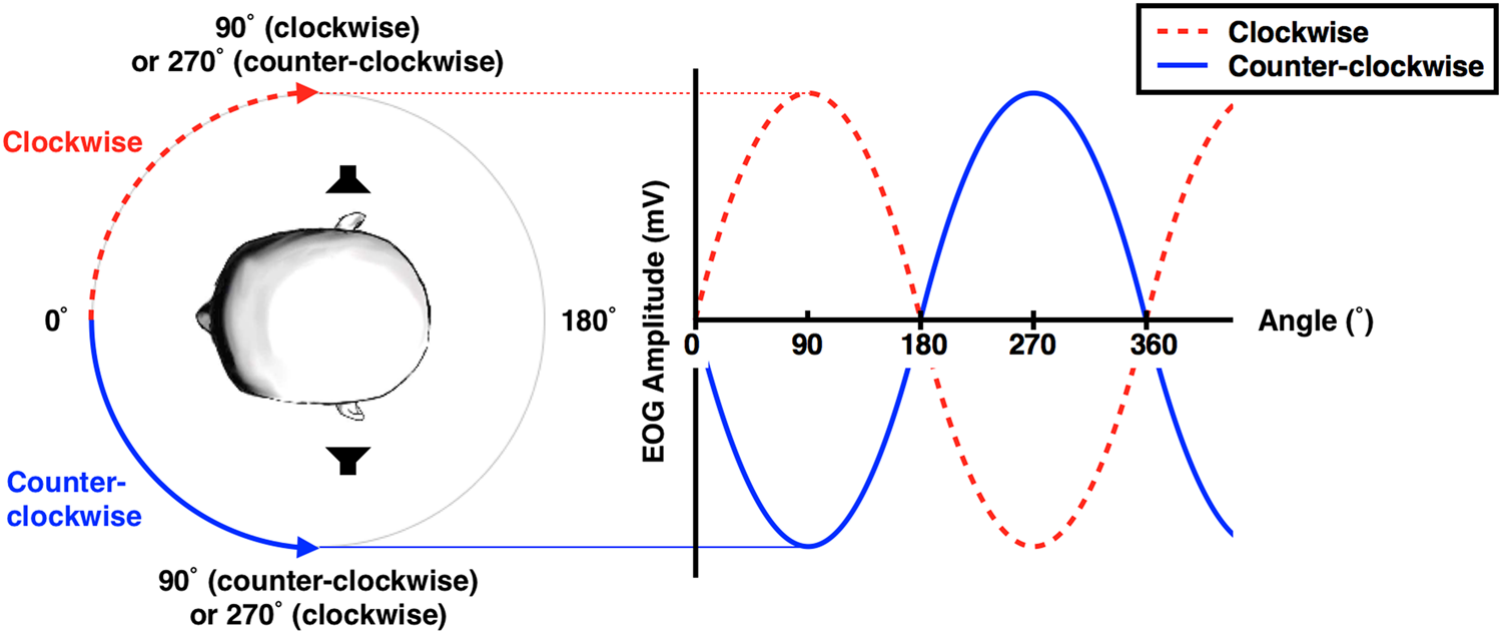
Theoretical horizontal EOG waveforms with respect to the rotating directions of auditory stimuli. Two sound stimuli spatially rotating either clockwise (CW) or counterclockwise (CCW) with different tone frequencies were simultaneously provided around the subject’s head with the subject’s eyes closed. Horizontal EOG shifts would be shown in different patterns with 180° phase difference, depending on which sound stimulus the subject was selectively attending to.

Taken together, it is highly required to develop a new AAC system addressing the limitations of conventional ones, which is easy to use, reliable, and does not require any calibration. In the present study, we propose a new EOG-based HCI paradigm for the binary yes/no communication in ALS patients, which has several strengths: 1) user’s voluntary eye movement is not required, 2) high accuracy is achievable without any training session, and 3) no calibration is needed. The proposed HCI uses horizontal EOG that is elicited by the autonomic eyeball response to a rotating sound source. A sinewave-like horizontal EOG waveform is generated by the auditory oculogyric reflex when a user is attending to a rotating sound. The main idea is to present a user with two sound sources rotating in directions opposite to each other, and instruct the user to selectively attend to one of the two auditory stimuli, based on the user’s intention. Since different EOG patterns would be generated based on the sound source on which the user directed increased attention to, the user’s binary yes/no intentions can be relatively easily recognised (see Fig. 1). This paradigm does not require any voluntary or conscious eyeball movement, but merely requires maintaining selective attention to one of the two sound sources, which is an easier task for patients with severe ALS than conventional EOG-based methods that require voluntary eye movements. To test the reliability and feasibility of the proposed method in practical situations, we recruited healthy subjects for offline/online experiments, and an ALS patient for online experiments.

## Results

### Sound stimuli

Thirteen healthy subjects (11 males and 2 females, 22–30 years old), including five who also participated in online/offline experiments of this study, were recruited to evaluate the suitability of the spatially rotating sound stimuli. Two songs with different tone and pitch (male voice and female voice) were played simultaneously, and were virtually rotated in opposite directions. After the suitability test experiment, two participants (15.4%) indicated that both songs were well-balanced. Five participants (38.5%) indicated that the sound stimulus was weighted towards the song with the male voice, while the rest (46.2%) thought that it was weighted towards the song with the female voice. In regard to the rotational speed and direction, eleven out of thirteen participants (84.6%) indicated that the rotational speed was adequate with clear rotational direction, either counter-clockwise or clockwise. One participant commented that the rotational speed was fast for him/her to follow the spatial movements of each song, and the other mentioned that he/she felt that both songs were virtually rotating in the clockwise direction. As a result, we concluded that the sound stimuli used for the experiment were not biased towards either song, and were optimised with appropriate speed and clear direction.

### Offline preliminary experiment

**Figure 2:**
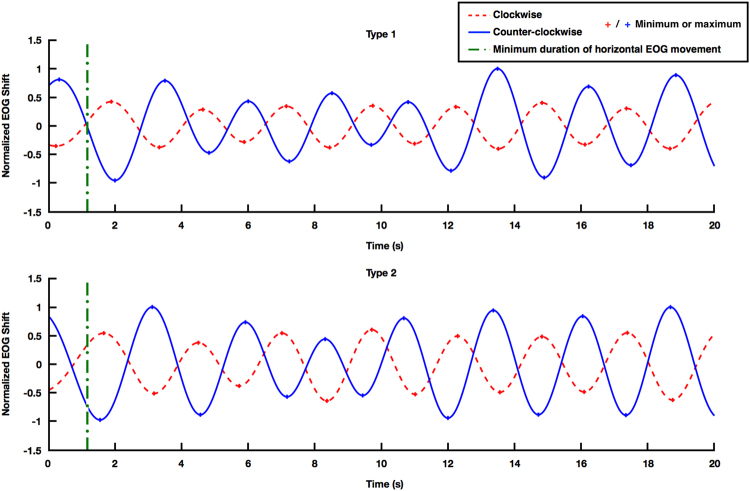
Two types of horizontal EOG waveforms. Each horizontal EOG waveform was assigned into one of two types based on the presence (Type 1, upper figure) and absence (Type 2, lower figure) of a peak at the very beginning of the task. Note that these data were plotted using averaged clockwise or counter-clockwise horizontal EOG waveforms of H3 (upper) and H1 (lower). The green vertical line in each figure represents the minimum horizontal eye movement duration (1.17 s in this study).

To develop an appropriate classification algorithm, we conducted a preliminary offline experiment (denoted as Exp1) with three participating subjects (H1–H3). Averaged waveforms of horizontal EOG signals recorded while each of the participants were selectively attending to one of the two songs rotating in clockwise or counter-clockwise directions yielded waveforms that were almost periodic, with phase differences between them of approximately 180° (see Fig. 2). Note that these EOG waveforms were generated by the participants’ autonomic eyeball responses to a rotating sound source rather than by their voluntary eye movements. Because it was necessary to minimise the time for detecting the user’s selective attention, we carefully observed the early changes in the EOG waveforms. We found that each horizontal EOG waveform could be assigned into one of two types, depending on (a) the presence (Type 1; upper figure of Fig. 2), or (b) absence (Type 2; lower figure of Fig. 2) of a peak at the very beginning of a task. This difference originated from the individual difference in the auditory oculogyric reflex speed. The actual horizontal EOG waveforms were different from the ideal waveforms depicted in Fig. 1 as the EOG signal was pre-processed using a bandpass filter and DC drift removal (see Supplementary Fig. S1). To design an algorithm that can estimate the binary intention of a user, regardless of the types of the EOG waveforms, the mean difference and the standard deviation between adjacent peaks (regardless of signs) in each participant’s EOG waveform were first evaluated, as summarised in Table 1. In this case, the mean difference indicates the duration of eye movement either from the left to right, or from the right to the left. The minimum duration of the horizontal eye movement was then defined as the mean difference of each subject subtracted by the corresponding standard deviation value. The averaged, minimum duration from the three participants was 1.17 s, which was then selected as an empirical guideline value for determining the types of EOG waveforms in additional studies. In other words, if a peak is present within the first 1.17 s after the onset of sound stimuli, the EOG waveform would be identified as Type 1. If an EOG waveform is identified as a Type 1 waveform, the EOG waveform can be transformed into a Type 2 waveform by eliminating the time period from the first data point to the first peak. We confirmed that this transform with an empirical value of 1.17 s was always successful at least in our experimental data. After the transformation, a single classification strategy could be applied. The classification method used in this study is described in detail in the Methods Section. Note that the use of different pre-processing procedures can lead to different EOG waveforms, when slightly modified guideline values and/or classification criteria might be required.

**Table 1:** Minimum duration of horizontal eye movement evaluated using the results of Exp1.

Subject	H1	H2	H3
Mean difference (s)	1.40	1.72	1.32
Standard deviation (s)	0.30	0.47	0.17
Minimum duration (s)	1.10	1.25	1.15
Average of minimum duration (s)	1.17		

The mean difference indicates the duration of eye movement either from the left to the right, or from the right to the left. The minimum duration of the horizontal eye movement was defined as the mean difference of each subject subtracted by the corresponding standard deviation value. The averaged minimum duration of the three participants was 1.17 s.

To find the optimal duration of each trial, we also tested different sizes of time windows spanning 6, 9, 12, 15, 18, and 20 s. Since the classification accuracy remained the same, sound stimuli spanning 6 s in duration were used for further online experiments. Note that window sizes less than 6 s yielded reduced classification accuracy when the current pre-processing method and the simple classification strategy were used.

### Online experiments

**Figure 3:**
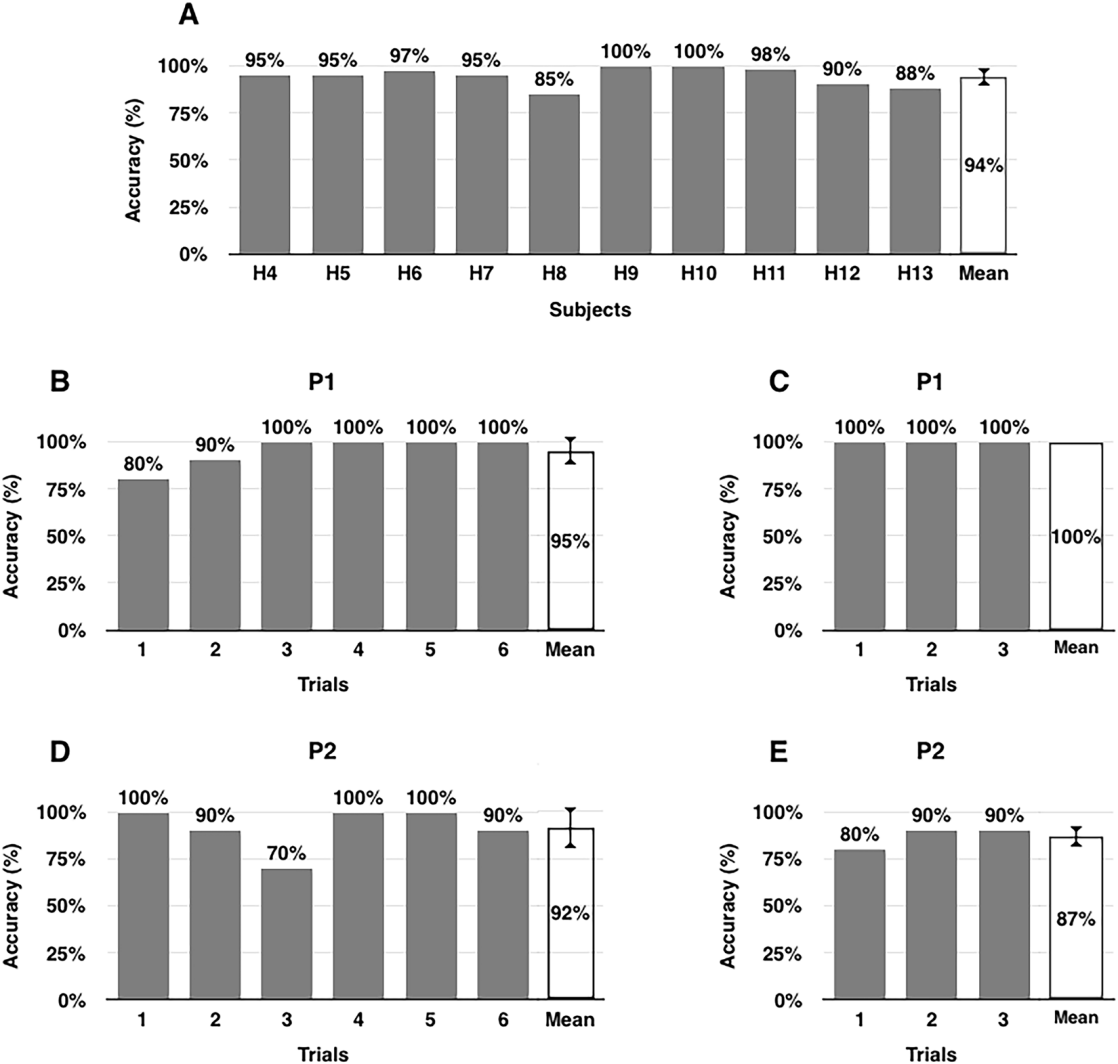
Online classification accuracy evaluated from Exp2 and Exp3. (**A**) Results of Exp2 (online) performed with 10 healthy subjects. The average classification accuracy was 94%. Nine out of ten healthy subjects yielded accuracies higher than 85%. (**B**) Results of Exp2 (online) with one ALS patient (P1). An average accuracy of 95% was reported. (**C**) Results of Exp3 (online with yes/no questions) with P1. The classification accuracy was 100%. (**D**) Results of Exp2 (online) with the other ALS patient (P2). An average accuracy of 92% was reported. (**E**) Results of Exp3 (online with yes/no questions) with P2. The classification accuracy was 87%.

To test the feasibility of our algorithm, we performed online experiments (denoted by Exp2) with ten healthy subjects (H4–H13), and two patients with ALS (P1–P2). The average accuracy of H4–H13 was 94% (Fig. 3A), while P1 and P2 completed the experiment with a classification accuracy of 95% (Fig. 3B) and 92% (Fig. 3D), respectively. The classification accuracies were much higher than the level of chance (approximately 62.5%) when the confidence level is 95%. Note that the chance level is approximately 62.5% when the number of trials in each class is 30, and the confidence level is 95%^33^.

To further demonstrate the practicality of the proposed paradigm, we conducted an additional online experiment (denoted by Exp3) with P1 and P2 using a yes/no questionnaire. The classification accuracy was 100% for P1 (Fig. 3C) and 87% for P2 (Fig. 3E), which further confirmed the feasibility of the proposed paradigm (please watch video clips showing the entire experimental procedure at https://youtu.be/CvCFtRUb59E and https://youtu.be/-cnqRkJD1Ao).

## Discussion

In this paper, we proposed an EOG-based HCI paradigm using two spatially rotating sounds and investigated its performance and practical usability through a series of online and offline experiments. In our paradigm, two sound stimuli were designed to spatially rotate in opposite directions—clockwise and counter-clockwise—and were simultaneously presented to the users when their eyes were closed. This approach was based on previous findings of human behaviour and physiological responses of 1) sound localisation^34^ and 2) selective auditory attention^35,36^.

Through the offline experiments (Exp1) with three healthy subjects, we found that most people with normal auditory function (all participants in our experiments) were able to localise the spatial location of rotating sound sources when they concentrated on a specific song over another, and their eyes followed the rotating sound source involuntarily. This horizontal eyeball movement can be readily detected using changes in the horizontal EOG waveform recorded from a pair of electrodes attached approximately 2.5 cm away from the lateral canthus of each eye. The horizontal EOG waveforms showed different patterns depending on the sound source that the participant selectively concentrated on (Fig. 1), and the participant’s selective attention could thus be reliably identified using the developed classification algorithm.

The online experiments with healthy subjects (Exp2) resulted in high classification accuracy. Nine out of ten healthy subjects achieved an accuracy that was higher than 88%. Only one subject (H5) yielded the relatively low accuracy of 85%. The reasons for this low performance may be attributed to tiredness and lowered concentration. Another possible reason could be that the participant had difficulties in getting accustomed to the system. Indeed, the classification accuracy was improved in later sessions: 60% on session 1, 80% on session 2, 100% on session 3, and 90% on sessions 4–6. Similar trends could also be observed in the results of an online experiment with a patient with ALS (P1). As shown in Fig. 3B, the classification accuracy was gradually enhanced as the session progressed.

In the online experiments with two patients with ALS (Exp2 and Exp3), it was shown that the proposed paradigm could successfully identify each patient’s yes/no intentions with high accuracy. Considering that a patient, P1, lost the control of voluntary blinking, and that could only move her eyes very slowly, our paradigm is expected to be potentially applied to the communication of some late-stage ALS patients. Nevertheless, one limitation of this approach is that it may not be used for the communication of patients who have severely impaired oculomotor functions. A number of case studies on ALS patients have reported a wide range of abnormalities in eye movements among the patients with ALS^37,38^. Indeed, we performed additional test trials with a male ALS patient who could not move his eyeballs to the right, and as a result, the classification accuracy was reported to be approximately equal to the random chance level (67.5%).

It is expected that the proposed AAC technology might be applied to some patients with ALS who only can use eye movement as a mean of communication (equivalent to LIS) and/or are starting to lose their eyeball control. Both ALS patients (P1 and P2) had originally used a camera-based eyeball mouse, but could not use it anymore at the time of the experiment owing to their impaired oculomotor function. Moreover, since she could not control her eyelids voluntarily, she could not communicate with others using any existing AAC devices. Even though her involuntary eyeball movements due to the rotating sounds were subtle and slow, the EOG signals were sensitive enough to identify her selective attention to the rotating sounds.

We recruited volunteers from one of the leading neuromuscular centres in South Korea; however, unfortunately, two patients were the only participants who were appropriate for our paradigm because we had difficulties in recruiting ALS patients with similar severity of symptoms from our limited patient pool. We hope to extend our experiment to a potentially broader range of participants, and further investigate the applicability of the proposed paradigm in future studies. Then, investigation on the relationship between the stage of the ALS disease and the achieved performance as well as the generalization across experimental sessions would be an interesting topic. In addition, we believe that other locked-in patient populations, other than the ALS population, would also benefit from our approach, which is also a promising topic that we want to pursue in our future studies.

Despite the limitations of our study, our experimental results highlighted several strengths of the proposed paradigm. First, the proposed paradigm could reliably identify the user’s selective attention without any training sessions. Most of the advanced AAC technologies need training sessions to collect training data for machine learning, or to help users become familiar with the systems^39^. Our system consistently showed high performance in most participants without any prior training sessions. Moreover, although our HCI paradigm used bio-signals generated by the actual movement of eyeballs, the users did not need any voluntary motor execution. All they needed to perform was the selective attention to a rotating sound source, which was more like a mental imagery task that required relatively lighter mental burden. More importantly, the performance of our HCI paradigm was better than that of another EOG-based HCI paradigm that requires voluntary horizontal eye movement^23^.

The goal of our study was to develop an auxiliary AAC system that can be potentially used as an effective communication tool of patients with severe ALS. The online experiments with our system exhibited high information transfer rates (ITRs) of 6.7256 bits/min (Exp2 with healthy subjects) and 5.7808 bits/min (Exp2 with ALS patients), and high average classification accuracies of 94% (Exp2 with both healthy subjects and ALS patients), but we still believe that the performance of our system can be further improved in future studies by increasing the overall classification accuracies and reducing the time needed for classification. It is expected that our paradigm would contribute as an alternative, or as a complementary communication option, for ALS and LIS patients who have impaired eye movement control.

## Methods

### Overall experimental design

This study comprised three experiments: one offline experiment (Exp1) and two online experiments (Exp2 and Exp3). All experiments used the same sound stimuli, but the duration of the presentation of the sound stimuli and the detailed experimental protocols were different. The aim of Exp1 was to confirm the feasibility of the proposed HCI paradigm, and to develop an appropriate classification algorithm. Exp2 and Exp3 were designed to evaluate the online performance of the suggested HCI paradigm.

### Participants

Thirteen healthy subjects participated in this study: three (2 males and 1 female, 22–24 years old, denoted by H1–H3) in Exp1 and ten (7 males and 3 females, 19–26 years old, denoted by H4–H13) in Exp2. None of them had any history of neurological or neuropsychiatric diseases that might otherwise affect the study results. All the healthy subjects had normal hearing and normal eye movement.

After completion of Exp2 with healthy subjects, we further explored the practicality of the proposed paradigm with ALS patients. We obtained lists of ALS patients from the Department of Neurology in Hanyang University Seoul Hospital, one of the leading neuromuscular centres in South Korea, and recruited two appropriate candidates for the experiment.

We visited a patient with ALS (female, 46 years old, denoted by P1) for Exp2 and Exp3 conducted on two separate days. We conducted Exp2 on the first visit and Exp3 on the second visit (four months after the first visit). The patient was diagnosed with ALS six years before the first experiment. She had been mechanically ventilated for three years, and was being fed using a gastrostomy tube. She scored 5 on the Korean version of ALS functional rating scale-revised (K-ALSFRS-R) that evaluates her functional status with the scale ranging from 0 (severe impairment) to 48 (normal functioning)^40,41^. A few years prior to the study, she had tried a camera-based eye tracking system as an alternative means of communication, but the system was no longer applicable after she lost control of her voluntary eye blinking. Full-time care was provided in her home. At the time of the experiment, she was able to slowly move her eyes horizontally and relied on a letter board with a partner-assisted scanning approach to communicate with her family members and a caregiver. The caregiver read out letters, words, or statements, and she expressed ‘select’ or ‘agree’ by looking either towards the left or the right.

The other patient (male, 55 years old, denoted by P2) participated in two online experiments – Exp2 and Exp3 – on the same day. He was diagnosed with ALS seven years before the experiments. He intubated an endotracheal tube, three weeks prior to the experiment date for mechanical ventilation. He was fed using a gastrostomy tube and scored 8 on K-ALSFRS-R. He had also tried a camera-based eye tracking system, which was unhelpful due to inaccuracy even though his oculomotor function including eye blinking remained relatively intact. Therefore, the only means of communication, at the time of the experiment, between his family members or a caregiver and him was a specially-designed letter board.

All participants provided written informed consent prior to the experiments. In regard to the ALS patients, P1’s husband and P2’s caregiver provided written informed consent on behalf of the patient. This study was approved by the institutional review board committee of Hanyang University Hospital, and conformed to the Declaration of Helsinki for the proper conduct of research on humans.

### Auditory stimuli

During the experiments, two songs with different tone frequencies (female voice rotating in the clockwise direction versus a male voice rotating in the counter-clockwise direction) were played simultaneously: the female song was ‘You Belong With Me’ by Taylor Swift, and the male song was ‘When You Gonna Learn (Digeridoo)’ by Jamiroquai. Each song was designed to spatially rotate around the subject’s head either in a clockwise or in a counter-clockwise direction with a rotational speed of 1/3 Hz (120°/s). The durations of the song play were 20 s for Exp1 and 6 s for Exp2 and Exp3. During the entire experiments, the auditory stimuli were delivered to the participant using a noise-cancelling headphone.

Before designing the experimental protocols, we surveyed thirteen healthy subjects, including five who also participated in Exp1 or Exp2, to evaluate the applicability of the spatially rotating sound stimuli based on the following three factors: 1) balance between two songs, 2) rotational speed, and 3) rotational direction of each sound.

### Data acquisition

**Figure 4:**
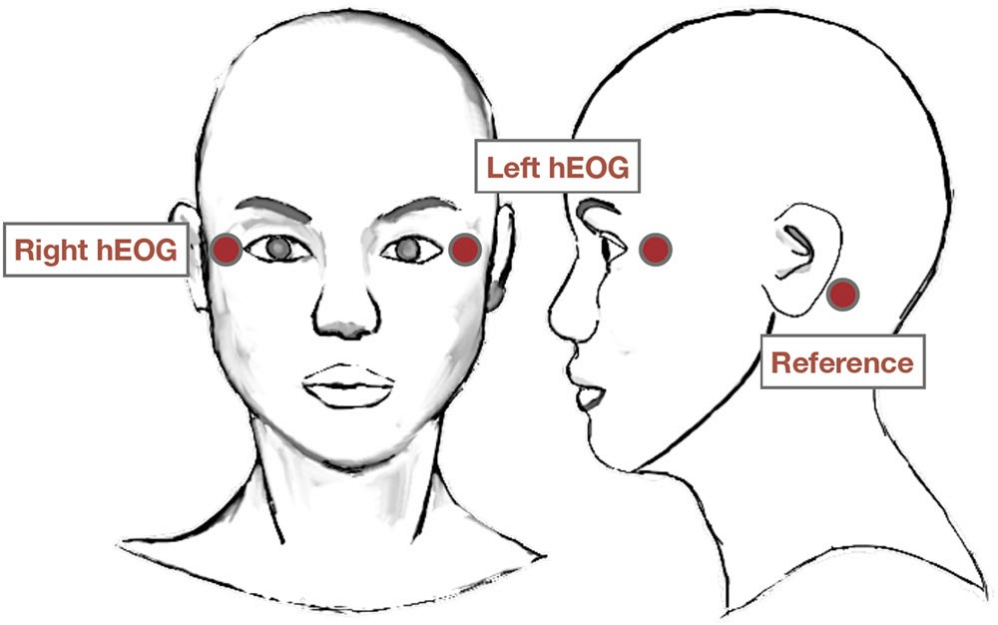
Electrode locations. In order to measure horizontal EOG signals, a pair of electrodes were attached approximately 2.5 cm away from the lateral canthus of each eye. These electrodes were referenced to the average of the potentials recorded from the left and right mastoid electrodes.

Horizontal EOG signals were recorded from a pair of electrodes attached at approximately 2.5 cm away from the lateral canthus of each eye (Fig. 4). The electrodes were referenced to the average position of the left and right mastoid electrodes. Data were acquired using a bio-signal acquisition system (BioSemi AciveTwo, Amsterdam, The Netherlands). The sampling rate was set at 2048 Hz. StimTracker (Cedrus Corporation, San Pedro, CA, USA) was used to mark the stimulus onset and offset. E-Prime 2.0 (Psychology Software Tools, Inc., Sharpsburg, PA, USA) was used to present instructions and auditory stimuli.

### Signal processing

The acquired EOG signals were processed using MATLAB R2012b (MathWorks, Natick, MA, USA). For pre-processing, the EOG data from each electrode channel were first down-sampled at a rate of 512 Hz. The horizontal EOG shift was calculated by subtracting the right EOG signal from the left EOG signal, and the DC offset (mean value over each epoch) was subtracted from the EOG shift. A fourth-order Butterworth band-pass filter with cut-off frequencies of 0.2 and 0.4 Hz was applied to the baseline-corrected EOG signal to better observe the eye movement following sound stimuli rotating at a rate of 1/3 Hz (see Supplementary Fig. S1).

In the feature extraction stage, the first local minima or maxima of the preprocessed EOG signals was determined. The slope of the line connecting the first data point and the first local minimum/maximum was then estimated. Note that the Type 1 EOG waveform should be transformed into a Type 2 waveform before the slope values were evaluated, as described in the Results Section. After the slope value was estimated, it could be readily identified which sound source a participant selectively concentrated on based on the sign of the slope. As shown in the example of the Type 2 EOG waveform in Fig. 2, the slope would be positive when he/she attended to a sound source rotating in the counter-clockwise direction, while the slope could be negative when he/she attended to a sound rotating in the clockwise direction.

### Paradigm

All experiments were conducted in a dimly lit room. Each participant was asked to concentrate on one of two rotating sound stimuli with the eyes closed, according to the instructions received (in Korean). All instructions and sound stimuli were presented through a noise-cancelling headphone. Each healthy subject was seated on a chair in a quiet room, while a participant with ALS laid in the supine position in her home environment.

**Figure 5:**
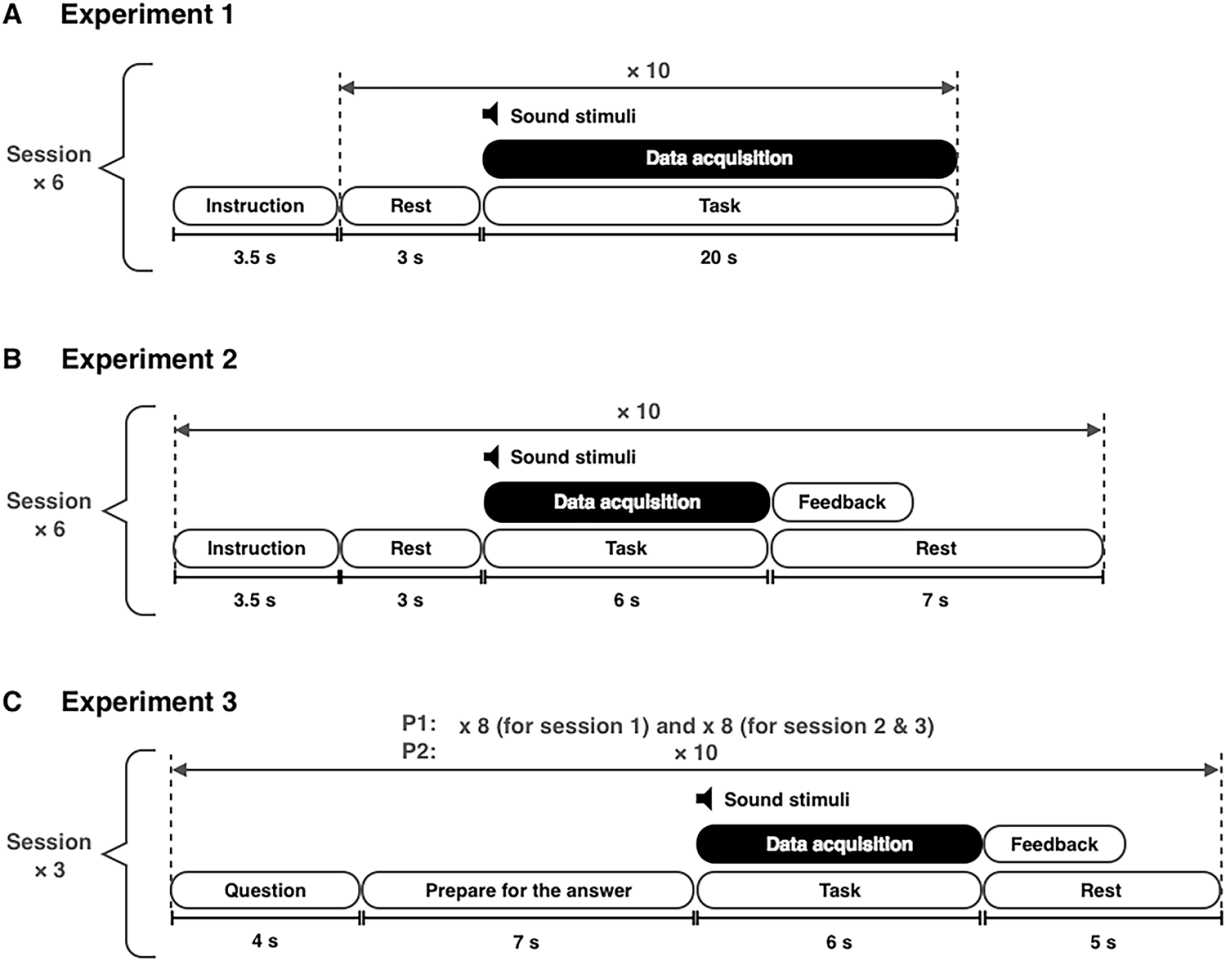
Schematics of experimental paradigms. (**A**) Exp1 (offline) consisted of six sessions with each comprising 10 trials. (**B**) Exp2 (online) consisted of six sessions with each comprising 10 trials. (**C**) Exp3 (online with yes/no questions) comprised (1) 10 trials on the first session, and 8 trials on the second and third sessions for P1 and (2) 10 trials on all three sessions for P2.

Exp1 consisted of six sessions with each comprising 10 trials. For each trial, each participant was asked to focus on a specific song for 20 s. A rest time period of 3 s was provided between trials, and approximately 3 min was provided between sessions (Fig. 5A).

Exp2 was performed in six sessions with each comprising 10 trials. For each session, either a clockwise or a counter-clockwise sound source, was randomly selected but counter-balanced. For each trial, each participant was asked to selectively focus on a specific song. Auditory feedback, either ‘female voice’ or ‘male voice’, was provided in Korean after the end of each trial, based on the classification results. A rest time period of 3 s and 7 s were given before and after each trial, respectively, and rest times of approximately 3 min were provided between sessions (Fig. 5B).

Exp3 was performed in three sessions, with 1) 10 trials in the first session and 8 trials in the second and third sessions for P1, and 2) 10 trials in all three sessions for P2. Unlike previous experiments, we gave the participating patients 26 (for P1) or 30 (for P2) different yes/no questions (see Table 2), and asked her to concentrate on the song with the female voice for answering ‘yes’, or the male voice for answering ‘no’. The response was presented as an automated voice for either ‘yes’ or ‘no’ right after each trial, based on the classification result. The preparation time for the answer was 7 s before every trial, the rest time was 5 s after each trial. Approximately 3 min were provided between sessions (Fig. 5C).

**Table 2:** List of twenty six (for P1) or thirty (for P2) questions used for Exp3 classified into three sessions.

Session - Trial	Questionnaire	Expected Result	
		P1	P2
**Session 1**			
1–01	Is your name “OOO”?	Y	Y
1–02	Is your nationality Korean?	Y	Y
1–03	Is your marital status “married”?	Y	Y
1–04	Do you live in Seoul?	Y	Y
1–05	Do you have children?	Y	N
1–06	Are you a male?	N	Y
1–07	Are you currently in a hospital?	N	Y
1–08	Are you 30 years old?	N	N
1–09	Do you have any pets at home?	N	N
1–10	Are you a student?	N	N
**Session 2**			
2–01	Can butterflies fly?	Y	Y
2–02	Is an apple a fruit?	Y	Y
2–03	Does one plus one equal two?	Y	Y
2–04	Is a piano an instrument?	Y	Y
2–05	Can dogs fly?	N	N
2–06	Are pencils edible?	N	N
2–07	Are dogs insects?	N	N
2–08	Are bears insects?	N	N
2–09	Are bears mammals?	N/A	Y
2–10	Are shells mammals?	N/A	N
**Session 3**			
3–01	Is a guitar an instrument?	Y	Y
3–02	Is the summer in Korea hot?	Y	Y
3–03	Are butterflies insects?	Y	Y
3–04	Can sharks fly?	N	N
3–05	Can cows fly?	N	N
3–06	Are butterflies plants?	N	N
3–07	Are grapes insects?	N	N
3–08	Are horses plants?	N	N
3–09	Are pigs mammals?	N/A	Y
3–10	Are cows mammals?	N/A	Y
Y: 12/N: 14			Y: 16/N: 14

The first session included 10 personal questions with known answers, and the second and third sessions contained basic, general knowledge, yes/no questions.

### Data availability

All relevant data are available at the following Figshare 10.6084/m9.figshare.6525164.v1.

## Electronic supplementary material

Supplementary Figure S1
