# Supplementary material for: Development of an electrooculogram-based human-computer interface using involuntary eye movement by spatially rotating sound for communication of locked-in patients
Source: Sci Rep. 2018 Jun 22;8:9505. doi: 10.1038/s41598-018-27865-5 (PMC6014992; doi:10.1038/s41598-018-27865-5)
Supplement: Supplementary file 1 — Supplementary Figure S1 [file 41598_2018_27865_MOESM1_ESM.pdf]

Supplementary Figure

Development of an electrooculogram-based human-computer interface using involuntary eye movement by spatially rotating sound for communication of locked-in patients

by Do Yeon Kim, Chang-Hee Han & Chang-Hwan Im

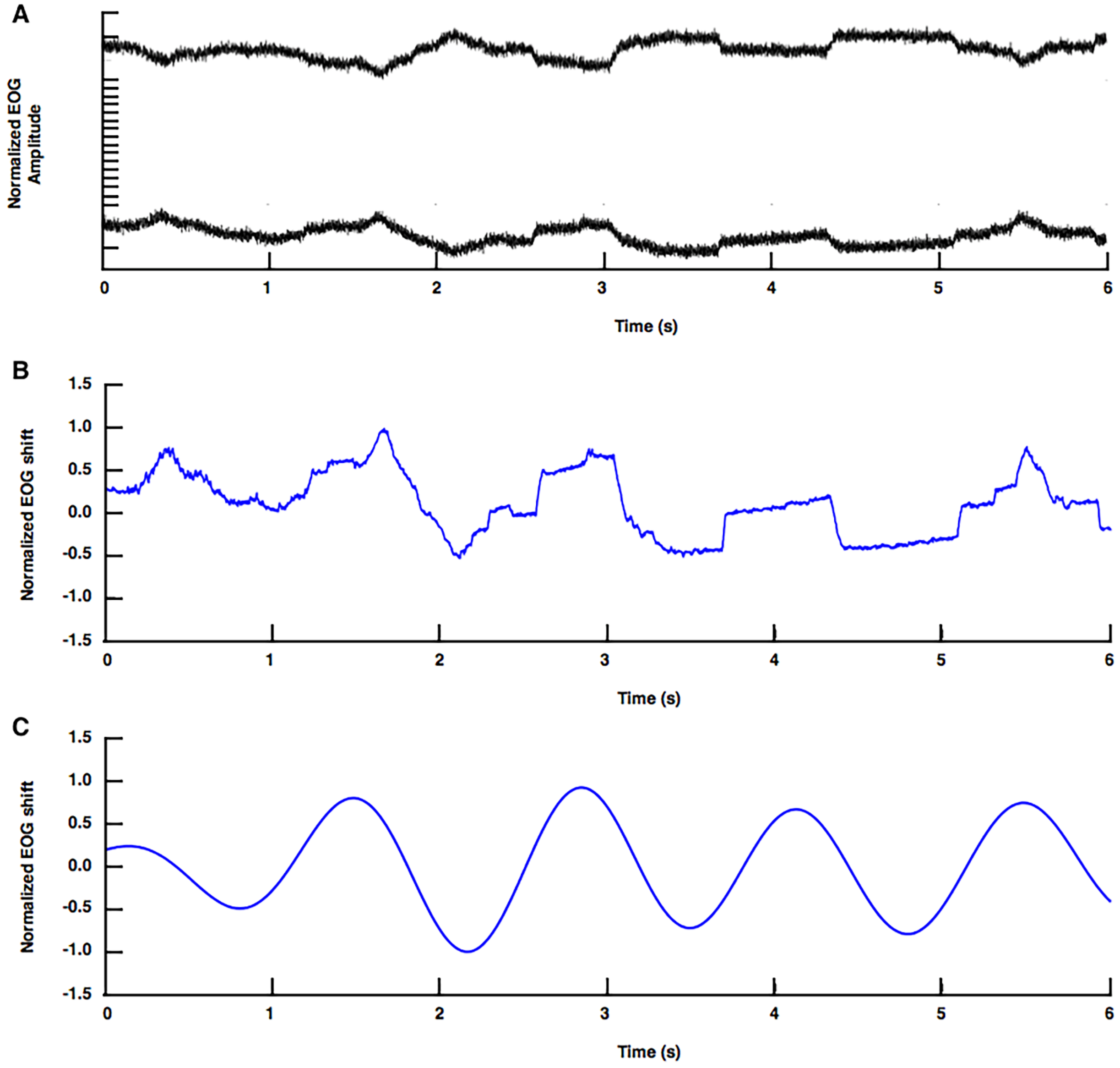

Supplementary Figure S1. An example of recorded EOG and its preprocessed signal during a task in Exp2. (a) Two recorded, unprocessed EOG signals: left EOG (bottom) and right EOG (top). (b) Normalized horizontal EOG shift obtained by subtracting right EOG from left EOG. (c) Normalized horizontal EOG shift after applying a fourth-order Butterworth zero-phase bandpass filter (0.2-0.4 Hz).
